# Harmful marketing by commercial actors and policy ideas from youth

**DOI:** 10.1093/heapro/daad149

**Published:** 2023-11-17

**Authors:** Maria Soraghan, Toyyib O Abdulkareem, Bethany Jennings, Juliet Akuamoah Boateng, Julissa Chavira García, Vaishali Chopra, Barsa Priyadarshini Rout, Kenechukwu Ujam, Sarah L Dalglish

**Affiliations:** CAP-2030, Institute for Global Health, University College London, 30 Guilford St, London WC1N 1EH, UK; NCD Alliance, Institute of Population Health, University of Liverpool, Brownlow Hill, Liverpool L69 7ZX, UK; CAP-2030, Institute for Global Health, University College London, 30 Guilford St, London WC1N 1EH, UK; Ghana NCD Alliance, 1 Hamilton St, Oyarifa, Accra 233, Ghana; Alianza Juvenil, 2403 Versailles Ct. McKinney, Texas 75070, USA; HRIDAY and Healthy India Alliance (India NCD Alliance), N-25, Green Park Extension, New Delhi-110016, India; HRIDAY and Healthy India Alliance Representative, N-25, Green Park Extension, New Delhi-110016, India; Association of Advocates Against Alcohol Harm in Nigeria, 109 Adegboyega Ago palace, Lagos, Nigeria; CAP-2030, Institute for Global Health, University College London, 30 Guilford St, London WC1N 1EH, UK

Commercial marketing, the set of practices carried out by commercial enterprises to promote sales or increase brand influence (Gilmore *et al.*, 2023), threatens the health and well-being of children and young people over the life course through the promotion of unhealthy products, habit formation and marketing techniques themselves (Guariguata and Jeyaseelan, 2019) (NCD Alliance & CAP-2030, 2023). The marketing of unhealthy products such as tobacco, alcohol, ultra-processed and/or high in saturated fat, free sugars, and/or sodium (HFSS) foods and non-alcoholic beverages, breastmilk substitutes and gambling products contributes to rising rates of noncommunicable diseases (NCDs), the leading cause of death and disability globally, with over three-quarters of the burden in low- and middle-income countries (World Health Organization, 2016) (World Health Organization, 2023). Globally, over two billion people under the age of 20 live with NCDs (Guariguata and Jeyaseelan, 2019). Corporations marketing unhealthy products reap enormous profits by targeting children and young people without regard for their well-being (Gilmore *et al.*, 2023). Tactics used by marketers to reach young consumers also have detrimental effects on mental health, for example, reducing self-esteem and altering body image (Strandgaard, 2014).

Policy action to combat harmful marketing, such as restrictions on commercials and advertisements, prohibition of data collection for marketing purposes and labelling laws is predicated not just on health harms, but also on human rights (Granheim *et al.*, 2019). Aggressive marketing of unhealthy products violates children’s and young people’s rights to health and healthy environments, nutritious food, accurate information, privacy and freedom from exploitation, as codified in the UN Declaration of Human Rights (1948); UN International Covenant on Economic, Social and Cultural Rights (1966), United Nations Convention on the Rights of the Child (UNCRC) (1989) and UN Guiding Principles on Businesses and Human Rights (2011), among others. Governments that have signed international agreements upholding these rights are duty-bound to regulate harmful marketing to prevent harm to children and young people.

## INVOLVING YOUTH IN POLICYMAKING

Many children and young people can be keenly aware of their exposure to and impacts of aggressive marketing and have policy ideas on how to reduce such harms. Although children in particular are rarely involved in policy discussions on the issue of harmful marketing, the UNCRC enshrines the right of children to meaningfully participate in decisions that affect them, including commercial regulation (United Nations, 1989). Notable exceptions can be found in the UK, where the youth-led organization Bite Back was instrumental in the 2021 campaign for stricter regulation on unhealthy food and beverage marketing to children, resulting in the adoption of an advertising ban to children (Sweney, 2021; Commons Chamber, 2021). In Ghana, a network of young scholars and advocates were heavily involved in implementation of the ‘Advocating for Health Project’, which led to the enactment of a sugar-sweetened beverage tax in 2023 (Advocating for Health, 2023).

Beginning in May 2023, NCD Alliance and Children in All Policies 2030 collaborated with an expert advisory group to produce a policy report on harmful marketing of NCD risk factors to children and young people. We reviewed harmful marketing tactics to children and young people and regulatory responses aimed at restricting such marketing, with input from 14 youth aged 15–30 years from a dozen countries via focus group discussions, written feedback on specific questions, and reviews of the policy report (NCD Alliance & CAP-2030, 2023). This editorial aims to showcase youth participants’ concerns about harmful marketing and policy suggestions, which informed the policy report and its recommendations.

## MARKETING CONCERNS OF YOUTH

### Mental health effects

Children and young people were concerned with mental health effects from marketing to a greater extent than has been represented in policy conversations thus far. In focus group discussions, there was consensus that marketing often has negative impacts on self-image and self-esteem. Several participants mentioned skin whitening products and how marketing preys on young girls’ views of beauty, confidence and worthiness:

We don’t think having tan skin or brown skin is attractive enough, and that’s what we’re being shown. You can’t be confident unless and until you have lighter skin. You can’t get a good job if you don’t have lighter skin. These are the types of advertisements shown to us. (Female, Age 15, Pakistan/France)

Other participants said mental health issues arising from marketing can lead to eating disorders and the hyper-sexualisation of young people. One participant thought companies purposefully exacerbate societal problems and ‘*specifically choose what’s most vulnerable… to children and teenagers [and] capitalise on this*’ (Female, age 16, Hong Kong/UK). Adolescents and young adults are particularly vulnerable to deceptive advertising around body image, including weight-loss and muscle-building dietary supplements, and emerging evidence suggests that there are major falsehoods in the marketing of these products (Hua *et al.*, 2021). While beauty and health products are not typically an industry of focus in conversations about harmful marketing, youth perspectives highlight the need to consider mental health harms arising from such marketing.

### Celebrity endorsements and Corporate Social Responsibility

In focus group discussions, children and young people spoke at length about the use of celebrity endorsements and corporate sponsorships to sell unhealthy products. One participant from Nigeria mentioned how Coca-Cola uses celebrities to promote its products to young people. While endorsements are well-known marketing tactic (APA, 2004), children and young people highlighted this as a major problem with a strong negative influence that remains under-regulated. While the participants did not use the term ‘Corporate Social Responsibility’ (CSR), several of their examples reflected existing evidence of companies using the guise of CSR to market their products and increase or improve brand recognition (Siahaya and Smits, 2021; Thomas *et al.*, 2023). A participant from Australia explained how after children’s sports games, athletes received McDonald’s vouchers. Another participant from Ghana recalled a sugar-sweetened beverage company sponsoring a diabetes campaign. And a young person from Vietnam mentioned how companies use social media influencers with large followings to advertise sugary drinks and snacks to teenagers. These tactics were seen as particularly harmful and ‘*disheartening*’ (Male, age 30, Nigeria) in lower-income settings where children may be more susceptible to marketing from admired celebrities and brand-recognized companies.

### Exploitation of vulnerable populations

Young people, especially children, are vulnerable consumers, as they do not fully understand the persuasive intent of advertisements, making them more susceptible to marketing pressures around unhealthy products and activities (Emond an Griffiths, 2020). Within this group, participants across countries gave examples of how marketing tactics prey on the most vulnerable children in each setting.

Children from very poor backgrounds are impressionable and lacking the necessary knowledge and exposure. [They] assume that what they see on billboards and screens is the way that life should be. It also does not help that junk food is generally cheaper and more accessible than healthy meals. (Male, age 30, Nigeria)

One participant mentioned how in Asian countries, formula milk companies target impressionable new mothers and push ‘*artificial and not nutritious’* milk, harming children at their *‘most vulnerable stage*’ (Female, age 16, Hong Kong/UK). Another participant who has lived in Asia and Europe spoke about how companies are using children’s data and violating their privacy for marketing purposes without their knowledge or full understanding. While there is good evidence that companies aggressively market in low-income settings and among vulnerable populations (UNICEF, 2023) (Brown *et al.*, 2023), the examples brought out by children and young people highlight the universal and exploitive nature of these tactics, suggesting policymakers need to do better at protecting these groups.

## YOUNG PEOPLE’S POLICY PRIORITIES FOR COUNTERING HARMFUL MARKETING

### Tighten regulation of ultra-processed and/or HFSS food marketing

Children and young people’s ideas for countering harmful marketing not infrequently reflected the expert consensus, however, they emphasized different priorities and were likely to call for more robust regulation and marketing bans. Several participants zoomed in on unhealthy food marketing:

I would end junk food marketing. This would take the spotlight off unhealthy food and create a food system with a bias towards health, where companies prioritise… healthy products. (Female, Age 17, United Kingdom)

Children and young people identified unhealthy food and beverage marketing as a top priority for policymakers to regulate, echoing recent calls by experts from the World Health Organization (2023), UNICEF (WHO/UNICEF, 2023), the 2020 WHO-UNICEF-*Lancet* Commission on children’s health (Clark *et al.*, 2020), among others, and recommended both the regulation of unhealthy food and the intentional promotion of healthy foods and lifestyles, aligning with existing literature (Freeman *et al.*, 2016). Youths’ calls for regulation, however, arguably went further than existing policy guidance, with one person saying, ‘*we must move beyond tobacco and alcohol as the evil ones and extend [regulation] to junk food*’ (Female, age 29, Ghana), with other participants sharing similar sentiments.

### Digital platforms are under-regulated

Pervasive digital marketing, directed at young people and using enticing tactics designed to target and appeal to youth audiences, was frequently brought up by children and youth participants, in line with evidence on the specific concerns of this type of marketing (Matos *et al.*, 2022). Youth participants said the digital space, namely social media, apps, and gaming applications, should be better regulated, with stronger rules that are both monitored and enforced. Several participants said digital platforms should be held accountable for allowing or delivering harmful marketing. Currently, digital spaces are primarily self-regulated, although early evidence has found this to be ineffective at reducing children’s exposure (Galbraith-Emami and Lobstein, 2022). Youths cited this gap in regulation, with one participant under the age of 18 describing being served an advertisement for e-cigarettes on a food delivery app, despite the app knowing her age. Other participants said platforms should be regulated through law, specifically mentioning current issues around data collection and privacy, in addition to age verification. Of note, Apple revoked its ban on data tracking of children after backlash from the private sector (Albergotti, 2019). Through their stories, it was clear that youths thought digital marketing restrictions are inadequate and regulation of digital platforms themselves (instead of just unhealthy commodity industries) should be a priority.

### Marketing ‘*gimmicks*’ and falsehoods

Participants discussed at length the need to regulate marketing ‘*gimmicks*’, commonly referred to as ‘power’ in marketing literature (i.e. the creative and convincing elements of marketing, designed to make an advertisement appealing) (World Health Organization, 2023). Using other words, participants said marketing power should be regulated to protect impressionable children and young people:

They are including characters, toys, and various other strategies which are very eye-catching for us youth. So [a policy] like ‘the gimmicks which are directed exactly to children’ should be restricted. (Female, Age 28, India)

Children and young people were concerned with the problem of dishonest and misleading marketing. Several participants mentioned a product that was falsely marketed as a cure for COVID-19 in India. Others brought up more traditional products like Diet Coke, e-cigarettes (‘vapes’) and flavoured tobacco or nicotine products that are marketed using perceived falsehoods about health claims, and misleading claims in the marketing of breastmilk substitutes. Although the problem of marketing exposure and power is recognized by experts, children brought an added dimension by focusing on misleading and false claims, which was perceived as an additional violation. Youths’ effort to draw attention to this issue is one demonstration of the value of engaging with them in policy discussions, as power tactics are known to be under-researched and under-regulated (World Health Organization, 2016) (World Health Organization, 2023).

## CONCLUSION

Children and young people are acutely aware of harmful marketing and its negative effects on health and well-being and are frustrated with the proliferation of dishonest and misleading marketing. Engaging with youth brought attention to regulatory priorities that have sometimes been overlooked in discussions amongst adults, such as the mental health impacts and body image issues associated with the beauty industry, the seemingly lawless atmosphere of marketing in the digital space, the power of celebrity endorsements and CSR to influence young minds, and the urgency of regulating ultra-processed food marketing. Children and young people’s clear call for comprehensive marketing regulation that is strictly enforced further buttresses attention to this issue from policy, public health and human rights experts.

Our research highlights how youth experiences and ideas can improve adults’ understanding of policy issues and solutions, however garnering children and young people’s meaningful participation can be challenging. Our process was limited to selected or voluntary participants, with discussions conducted in English and relying on internet connection, skewing representation towards higher-resourced groups. Yet there is growing momentum to include children in decision-making despite challenges in doing so in a representative, effective and safe manner (Forde *et al.*, 2020). Our experience suggests three practical lessons. First, policy processes should engage children and young people from the onset, the problem definition stage, and not as an afterthought or add-on once the policy process is underway. Second, it is helpful to provide varied forums and participation methods to accommodate a range of children and young people’s engagement styles and developmental abilities, such as group discussions, written responses to questions, opportunities to review written outputs and inclusion in the communication of policy decisions. Third, it is critical to follow through on engagement by communicating how inputs are being used and to what end. Policymakers must recognize that children and young people are valuable stakeholders in decision-making processes and commit to their inclusion as equal co-creators in all decisions that concern their well-being, including in the regulation of harmful commercial marketing.
